# Insights from an online survey: Veterinary surgeons' antibiotic practices in ophthalmic surgery in Germany

**DOI:** 10.1111/vop.13300

**Published:** 2024-11-11

**Authors:** Claudia Busse, Anne Raab, Lothar Kreienbrock, Holger Andreas Volk

**Affiliations:** ^1^ Department of Small Animal Medicine and Surgery University of Veterinary Medicine Hannover Hannover Germany; ^2^ Department for Biometry, Epidemiology and Information Processing University of Veterinary Medicine Hannover Hannover Germany

**Keywords:** antibiotics, One Health, ophthalmic surgery, prophylaxis, surgical site infection, survey

## Abstract

**Purpose:**

To investigate antibiotic usage practices in ophthalmic surgeries in Germany.

**Materials and Methods:**

An online questionnaire was sent to veterinary surgeons (general veterinarians and veterinarians with additional qualification in ophthalmology) inquiring about their antibiotic preferences, administration methods, and factors affecting antibiotic usage in ophthalmic surgical procedures.

**Results:**

A total of 417 questionnaires were analyzed. Postoperative antibiotics (systemic/topical in percent when used) were used all or most of the time by 69% of veterinarians following enucleation (99/6), by 62% after eyelid surgery (54/69), by 68% after third eyelid (TEL) surgery (19/92) and by 80% after keratectomy (6/99). The most commonly used systemic antibiotic was amoxicillin with clavulanic acid and the most commonly used topical antibiotic was chloramphenicol.

WHO “watch‐group” antibiotics were infrequently administered systemically but frequently utilized topically; including in 13% of eyelid surgery, 15% of TEL surgery, and 35% of keratectomies. Factors influencing antibiotic use included fear of complications (67%), personal experience (63%), diagnostic uncertainty (21%), and owner expectations (9%). Participants following institutional guidelines used fewer antibiotics in enucleations (*p* = .002) and were less likely to choose fluoroquinolones post‐eyelid surgery (*p* = .044).

**Conclusion:**

The potential for reducing antibiotic use following ophthalmic soft tissue surgery is significant. Addressing barriers such as concerns about postoperative complications and the reliance on individual clinical experience, the implementation of standardized guidelines could facilitate a shift toward more judicious antibiotic practices.

## 1 INTRODUCTION

The use of antibiotics to prevent secondary bacterial infections is a widespread practice in veterinary ophthalmology. Antibiotics are used in both extra and intraocular surgeries. However, the use of antibiotics, especially in prophylaxis has been increasingly questioned in recent years, considering that they may not offer exclusive advantages.^1^ Disadvantages include potential side effects, such as discomfort or a delay in corneal epithelial wound healing, a negative impact on local or distant microbiomes as well as the development of antimicrobial resistances.^1^, ^2^

Antimicrobial resistance has emerged as a major concern, ranking amongt the top 10 global threats identified by the Word Health Organization (WHO).^3^ The WHO has also divided antibiotics in access, watch, and reserve antibiotics, to provide an indication of the appropriateness of antibiotics use.^4^ The development of antimicrobial resistance also poses a significant challenge in veterinary ophthalmology with up to 38.9% of multidrug‐resistant bacteria reported in patients with infectious keratitis.^5^ As veterinarians, it is imperative to actively participate in the One Health movement and critically evaluate our antibiotic usage.^6^, ^7^ A variety of guidelines governing the use of antibiotics across different body systems have been published by reputable institutions such as the World Veterinary Association, the World Organization for Animal Health, the directorate‐general for health and food safety of the European Union or the Federation of European Companion Animal Veterinary Association.^7^, ^8^, ^9^ However, none of these guidelines provides recommendations specifically tailored to the use of antibiotics in ophthalmic patients.

To promote a more responsible use of antibiotics in veterinary ophthalmology, it is essential to understand current practices. Therefore, the aim of this study was to investigate how antibiotics are used in veterinary ophthalmology in Germany. The data serve as a starting point to identify areas with potential to reduce and replace antibiotics without compromising outcomes for patients, ultimately making treatments in veterinary ophthalmology better and more sustainable.

## 2 MATERIALS AND METHODS

### 2.1 Study design and questionnaire implementation

A web‐based online questionnaire (LimeSurvey: An Open‐Source survey tool, LimeSurvey GmbH, Hamburg, Germany) was constructed to collect information on the use of antibiotics in ophthalmic patients as well as on different aspect surrounding this topic. Here we present only data of the specific part of the questionnaire that focuses on the use of antibiotics to prevent secondary bacterial infections in association with ophthalmic surgeries.

Target participants were veterinary surgeons with or without further qualification in veterinary ophthalmology in Germany who treat dogs and cats with ophthalmic conditions. Promotion of the questionnaire was through direct email requests to veterinary surgeons, indirect email requests through the Veterinary Chambers of different federal states and different German veterinary associations, distribution at a variety of veterinary conferences and through the monthly journal of the German Federal Chamber of Veterinarians as well as through the homepage and Facebook account of the university.

Data were collected between March 2022 and December 2022. The questionnaire included questions on demographic data, the use of peri‐ and postoperative antibiotics in different surgeries and potential factors, which may influence the decision‐making process when using antibiotics. Surgical procedures included enucleation, eyelid surgery, third eyelid (TEL) surgery and keratectomy. The questionnaire did not specifically address if this was for planned uninfected procedures or for all procedures including infected or traumatic cases that required surgery.

Participants were initially asked if they had enough experience with a certain procedure to give a meaningful answer, otherwise they were guided to the next question. Participants could choose one or more drugs from a list of systemic and topical antibiotics, which for topical medication also included examples of commonly available ophthalmic drugs for easier recognition as shown in Table 1. In the table WHO watch‐group antibiotics are highlighted for the readers information. Common combination products were also included. There was also the opportunity to free‐write if the required drug was not listed. Due to legislation in Germany, the use of compounded drugs is largely prohibited. Veterinarians have the choice of using drugs that are licensed in the European Union for the animal species they are treating or have to specifically justify the use of non‐veterinary products in individual case scenarios. The latter usually means using medication licensed for humans. Perioperative antibiotics were defined as an intravenous application 30–60 min before the first incision and repeated every 90 min until final would closure.

**TABLE 1:** List of antibiotics and antibiotic groups participants were able to choose from in each selected case scenario.

Systemic antibiotics	Topical antibiotics	Perioperative antibiotics
Amoxicillin	Chloramphenicol	Amoxicillin
Amoxicillin–clavulanic acid	**Chlortetracycline**	Amoxicillin–clavulanic acid
First generation cephalosporins	**Ciprofloxacin**	First generation cephalosporins
**Third generation cepalosporins**	Fucidic acid	
Clindamycin	Gentamicin	
Doxycycline	**Moxifloxacin**	
**Marbofloxacin**	Neomycin	
**Enrofloxacin**	Polymyxin‐B	
**Pradofloxacin**	**Ofloxacin**	

*Note*: Perioperative antibiotics were limited to eyelid and third eyelid surgery. WHO “Watch” antibiotics are written in bold.

The data recruitment and the questionnaire complied with the current Data Protection Directive of the European Union and Germany and was authorized by the data protection officer of the University of Veterinary Medicine Hannover Foundation.

### 2.2 Data analysis

Statistical analysis was performed using SAS software (SAS Enterprise Guide 7.1.). Associations between two characteristics were analyzed and calculated using Pearson's chi‐square homogeneity test and Fisher's exact test, using *p*‐values <0.05 as being statistically significant. Given the lack of experience of participants with certain surgeries, total sample sizes vary between case scenarios. Participants could choose more than one antibiotic as possible treatment options, which means that the percentages of antibiotics listed exceeds 100%.

## 3 RESULTS

**FIGURE 1:**
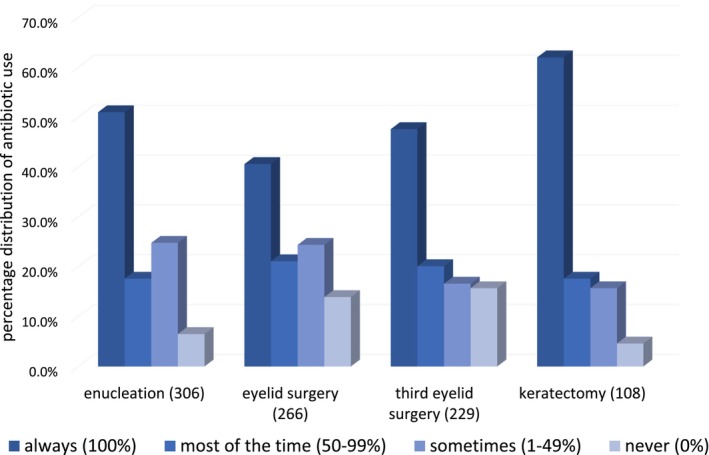
Percentage distribution of postoperative antibiotic use by German veterinary surgeons for four common ophthalmic surgeries. The number of respondents for each procedure is indicated in parenthesis next to the surgery.

**FIGURE 2:**
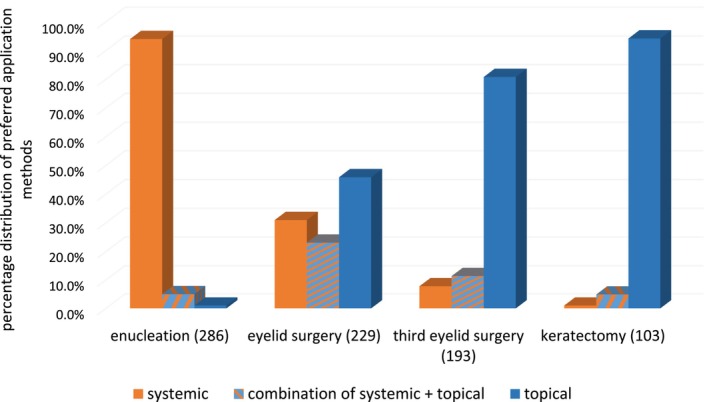
Percentage distribution of preferred application methods (systemic and/or topical) used by participating small animal practitioners in Germany for four common ophthalmic surgeries. The number of respondents is indicated in parentheses next to each case example.

We analyzed 417 replies, of which 311 were fully completed. The number of participants per case scenario varied as answers were only given if participants felt they had experience with a given condition. Data are shown in Table 2, and Figures 1 and 2.

**TABLE 2:** Postoperative topical antibiotic selection by German veterinary surgeons for surgical procedures in percent.

	Participants	Tetracycline	Steroid‐AB	Phenicol AB		Aminoglycoside AB		Aminoglycosid + peptide‐AB	Fluoroquinolones			Other (%)
	*n*	CTZ (%)	FUS (%)	CAP (%)	CAP + DEXA (%)	GENTA (%)	NEO + 1 (%)	NEO/POLY (%)	MOX (%)	OFXC (%)	CFZ (%)	
Enucleation	17	**6**	6	35	6	53	6	0	**0**	**29**	**0**	0
Eyelid surgery	158	**34**	13	51	5	32	6	2	**1**	**11**	**1**	6
Third eyelid surgery	178	**28**	14	43	7	35	3	4	**2**	**14**	**1**	4
Keratectomy	102	**28**	3	67	0	32	0	0	**9**	**28**	**6**	8

*Note*: More than one choice per condition was possible. “*n*” denotes the number of participating veterinarians for each surgical procedure. WHO “Watch” antibiotics are highlighted in bold.

Abbreviations: CAP, chloramphenicol; CAP + DEXA, chloramphenicol + dexamethasone; CFZ, ciprofloxacin; CTZ, chlortetracycline; FUS, fucidic acid; GENTA, gentamicin; MOX, moxifloxacin; NEO + 1, neomycin + hydrocortisone acetate + lidocaine hydrochloride + vitamin A + retinol; NEO/POLY, neomycin + dexamethasone + polymyxin‐B‐sulfate; OFXC, ofloxacin.

### 3.1 Participants' demographic data

Participants worked either in veterinary practices with one to five veterinarians (*n* = 235; 56%), more than five veterinarians (*n* = 57; 14%), animal hospitals with 24 h. patient care (*n* = 63; 15%) or in single‐vet practices (*n* = 55; 13%). Few veterinarians worked at more than once practice (*n* = 7, 2%). Most participants were general veterinary surgeons (71%), some (14%) were specialized in areas other than ophthalmology and the remaining participants were either holding the national certificate in ophthalmology (7%), diplomates of the ECVO or ACVO (1%) or training for either of these qualifications in ophthalmology (9%).

### 3.2 Antibiotic use in surgical procedures

#### 3.2.1 Enucleation

Out of 306 participants that had experience with enucleations, two thirds (69%) reported to use antibiotics either all the time (51%) or most of the time (18%). One quarter (25%) used antibiotics occasionally while 7% never used antibiotics in enucleations. Systemic antibiotics were most commonly utilized (94%), with amoxicillin with (94%) or without (14%) clavulanic acid being the most commonly used agents (Table 3). Fluoroquinolones were listed as systemic treatment option by 10% of the participants.

**TABLE 3:** Postoperative systemic antibiotic selection by German veterinary surgeons for specified surgical procedures.

	Participants	Aminopenicillin (%)		Cephalosporine		Lincosamid	Tetra‐cycline	Fluoro‐quinolone (%)
	*n*	Amoxicillin	Amoxi clav	1st generation	3rd generation	Clindamycin	Doxycycline	
Enukleationen	283	14	94	5	**2**	2	1	**10**
Eyelid surgery	124	17	88	3	**1**	1	1	**3**
Third eyelid surgery	37	35	68	0	**3**	3	0	**3**
Keratectomy	6	17	100	0	**0**	17	0	**0**

*Note*: More than one choice per condition was possible. “*n*” denotes the number of participating veterinarians for each procedure. WHO “watch” antibiotics are highlighted in bold.

Abbreviation: Amoxi clav, amoxicillin clavulanic acid.

#### 3.2.2 Eyelid surgery

62% of the 266 participants with experience in eyelid surgery used antibiotics either always (41%) or most of the time (22%), while 38% used antibiotics sometimes (24%) or never (14%). When antibiotics were used, 46% of participants choose systemic antibiotics alone, 23% used a combination of systemic and topical antibiotics and 31% topical antibiotics alone. Twenty‐five percent of the participants gave perioperative antibiotics, mainly amoxicillin with (53%) and without (25%) clavulanic acid (Table 4). The most commonly used postoperative systemic antibiotic was amoxicillin with (88%) or without (17%) clavulanic acid (Table 3). Antibiotics listed in the “watch‐group” of the WHO included third generation cephalosporins (1%) and fluoroquinolones (3%). Systemic antibiotics were mostly given for the duration of up to 1 (64 %) or 1–2 weeks (36%). The most commonly used topical antimicrobials included chloramphenicol (51%), chlortetracycline (34%), and gentamicin (32%) (Table 2). At least one topical fluroquinolone (ciprofloxacin, ofloxacin, or moxifloxacin) was listed as a possible treatment choice by 13% of the participants. Topical antibiotics were mostly recommended three times daily (59%) or more (25%) (Table 5).

**TABLE 4:** Perioperative antibiotic selection by German veterinary Surgeons for specified surgical procedures.

Surgical procedure	Participants	Aminopenicillin (%)		Cephalosporine (%)		None (%)
	*n*	Amoxicillin	Amoxicillin + clavulanic acid	1st generation	3rd generation	
Eyelid surgery	229	6	13	4	**0,4**	75
Third eyelid surgery	193	5	11	2	**0**	80

*Note*: More than one choice per condition was possible. “*n*” denotes the number of participating veterinarians for each procedure. WHO “watch” antibiotics are highlighted in bold.

**TABLE 5:** Period of postoperative antibiotic treatment by German veterinary surgeons for surgical procedures.

Surgical procedure	Participants	Frequency of topical application (%)				Duration of topical treatment (%)			Participants	Duration of systemic treatment (%)		
	*n*	1×/day	2×/day	3×/day	>3×/day	<1 weeks	1–2 weeks	>2 weeks	n	<1 week	1–2 weeks	>2 weeks
Enucleation	17	6	24	41	29	12	65	24	283	58	41	1
Eyelid surgery	158	3	13	59	25	44	53	3	124	64	36	1
Third eyelid surgery	178	1	14	55	30	52	46	2	37	70	30	0
Keratectomy	102	1	7	41	51	17	58	26	6	17	83	0

*Note*: “*n*” denotes the number of participating veterinarians for each procedure and frequency or type of application.

#### 3.2.3 Third eyelid surgery

Of the 229 participants with experience in TEL surgery, 68% used antibiotics always (48%) or most of the time (20%) in association with surgical procedures in this area, while 32% used antibiotics sometimes (17%) or never (16%). When antibiotics were used, 8% of the participants chose to use them only systemically, 11% combined systemic and topical application, while 81% chose a solely topical application. Perioperative antibiotics were given by 20% of the participants, mainly amoxicillin with (60%) and without (24%) clavulanic acid (Table 4). The most used postoperative systemic antibiotic was amoxicillin with (68%) or without (35%) clavulanic acid (Table 3). Antibiotics listed in the “watch‐group” of the WHO included third generation cephalosporins (3%) and fluroquinolones (3%). Systemic antibiotics were given for less than 1 week (70%) or for 1–2 weeks (30%). Topically, the most commonly used antimicrobials following TEL surgery included chloramphenicol (43%), gentamicin (35%), and chlortetracycline (28%) (Table 2). At least one topical fluorquinolone was listed as treatment option by 15% of the participants. Topical antibiotics were mostly applied 3× daily (55%) or more (30%) for either less than a week (52%) or 1–2 weeks (46%) (Tables 4 and 5).

#### 3.2.4 Keratectomy

Of the 108 participants performing keratectomies, 80% used antimicrobials either always (62%) or most of the time (18%) while 20% used them sometimes (16%) or not at all (5%). When antimicrobials were used, 1% of participants used systemic antibiotics alone, 94% chose topical antibiotics and 5% combined topical and systemic application. When a systemic antibiotic was chosen, it was always amoxicillin and clavulanic acid (100%) which was mostly used for 1–2 weeks (83%). Topically, the most commonly used antibiotics were chloramphenicol (67%), gentamicin (32%), chlortetracycline (28%), and ofloxacin (28%). At least one topical fluoroquinolone was listed as a treatment choice by 35% of the participants. Topical antibiotics were given 3× daily (41%) or more (51%) for 1–2 weeks (58%) or more than 2 weeks (26%) (Tables 4 and 5).

### 3.3 General questions

Out of 311 participants, 24% stated to be very or at least a little bit concerned about the increase of antimicrobial resistance, while most of the participants (72%) were not (53%) or mostly not (19%) concerned that antimicrobial resistance may become a problem. In addition to the clinical presentation, reasons for the use of systemic antibiotics as reported by 311 participants, were fear of complications (67%) and their own experience (63%). Sometimes diagnostic insecurity (21%) and owner expectation (9%) were also reported to influence the decision to use antibiotics. Exceptional circumstances for the use of systemic antibiotics included the inability of owners to apply topical medication (71%), concerns about secondary bacterial infections (53%), aggressive patients (53%), and time restraints of the owners, which prohibit adequate topical medication (26%). Almost one third of the participants (30%) had official guidelines on the use of antimicrobials in their work place, while the majority of participants (70%) did not. Participants that follow official guidelines by their institution used significantly less antibiotics in enucleations (*p* = .002) and were less likely to choose topical fluoroquinolones following surgical procedures of the eyelids (*p* = .044). The presence of institutional guidelines did not significantly influence the use of antibiotics in the other procedures.

## 4 DISCUSSION

To the authors knowledge, this is the first overview on the use of antibiotics in small animal ophthalmic patients in Germany. The survey confirmed that antibiotic use was considered currently as a major tool to prevent surgical site infections (SSIs) following surgical procedures. Antibiotics were utilized topically or systemically by the majority of veterinarians in all of the given procedures; including enucleations, eyelid surgery, TEL surgery, and keratectomy.

As in most online surveys the limitations of our study were the restrictions to the bias from nonresponse and under‐coverage of the entire study population, namely the German group of veterinarians treating animals with medical indication for ophthalmic surgery. Also, in certain scenarios surgeries may be performed in the face of infection, which will influence the choice to use an antibiotic or not. Since this was not specified in the question this may have influenced the answers of the participants. It is also important to notice that the selection of antibiotics was constrained by the range of licensed products available at the time, which were restricted by EU legislation and availability has changed since then. Also, some of the antibiotics were combined with other active ingredients, which will have influenced clinicians' choices. The use of perioperative antibiotics in enucleations was not part of the survey. This is unfortunate since this procedure is often performed by soft tissue surgeons. The use of antibiotics in enucleations may therefore be more in line with recommendations for soft tissue procedures, where the sole use of perioperative antibiotics is largely the modality of choice.^10^

SSIs are infections caused at time of or post‐surgery.^10^, ^11^ SSI rates in clean surgical wounds in small animal practice are reported between 0.8% and 4.8%. In dogs, they are mostly caused by staphylococci, especially *Staphylococcus (S.) pseudintermedius*, with a recent increase in methicillin and multidrug resistance.^10^, ^12^ Reported risk factors for SSIs in small animal procedures include hypotension, anesthesia time, clipping of hair, duration of surgery, patients' body temperature, endocrine disorders, and the number of people in the operating theater.^10^, ^13^ Mounting evidence suggests that the use of systemic antibiotics does not have a beneficial effect on SSI rate in clean and clean‐contaminated soft tissue, neuro‐ and orthopedic surgeries in dogs and cats.^13^ A multi‐centered study by Stetter et al. shows a great variation in perioperative antimicrobial use between countries. In Germany perioperative antibiotics were used in 83% of surgical procedures compared to the Scandinavian countries with 16%–33%. Nonetheless, the SSI rates did not significantly differ between these countries.^13^ This compelling evidence should prompt us to question the extensive use of antibiotics in ophthalmic soft tissue surgery.

Prophylactic postoperative antibiotics following enucleation did not reduce the overall risk of SSIs in another retrospective study with 280 dogs.^17^ In the same study, placing an orbital implant was not associated with an increased risk for infection. In the dogs where a SSI was identified with a positive culture, over half grew multidrug‐resistant organisms, which would not have responded to routine postoperative antibiotics.^17^ A retrospective study from the physician ophthalmology literature with over 480 procedures suggests lower infection rates in enucleations and eviscerations performed without any perioperative intravenous or postoperative oral antibiotics when compared to case series where antibiotics were given.^18^ The authors suggested that peri‐ and postoperative antibiotics in enucleations and eviscerations may not be indicated in humans.^18^

In eyelid surgery almost half of the veterinary surgeons chose systemic postoperative antibiotics and two thirds chose topical antibiotics. In human ophthalmology, infection rates following eyelids surgery are low (1.6%–2.3%) and not giving systemic antibiotics is not associated with increased SSI rates.^19^, ^20^ Depending on the surgical procedure; use of topical antibiotics in eyelid surgery should be questioned, since it is unclear if antibiotics applied to the ocular surface actually reach the surgical site at the periocular skin at an adequate concentration. In humans SSIs did not differ with or without the use of topical chloramphenicol.^19^

In TEL surgery, two thirds of veterinarians use antibiotics always or most of the time. Information regarding SSIs for clean surgeries of the conjunctiva are not available in the literature and were not listed amongst complications following different surgical techniques for the replacement of the TEL gland.^21^, ^22^, ^23^ If infections occur, the pathogens are likely to originate from the natural flora of the ocular surface. The low bacterial biomass of the conjunctiva is composed of a wide range of bacterial species.^24^, ^25^, ^26^, ^27^, ^28^, ^29^ The effect of antibiotics such as ciprofloxacin, chloramphenicol and neomycin‐polymyxin‐bacitracin on the composition of the conjunctival bacterial flora has been investigated, and authors found no significant effect.^29^, ^30^ This is usually used to argue that the use of topical antimicrobial agents has no detrimental effect on the microbiome; however, it also puts in question the use of topical antibiotics. If antimicrobials do not change the composition of the bacteria that may potentially be involved, do they actually prevent SSIs on the ocular surface? It is important to be aware that although the composition of the bacterial DNA may not be measurably modified, the antimicrobial susceptibility of the bacteria is in fact changed and may lead to treatment challenges should an infection occur later.^2^, ^5^, ^31^ This is a phenomenon that is also well reported in human ophthalmology.^32^, ^33^ Given that SSIs of clean conjunctival surgery have not been reported, the risk should be considered as very low.

Evidence suggests that the extensive use of peri‐and postoperative antibiotics in ophthalmic soft tissue surgery should be discontinued.^13^, ^14^, ^15^, ^16^, ^17^ The sole use of intravenous perioperative antibiotics may be considered initially. It is essential to bear in mind that controlling other known risk factors, will further lower the SSI rate. Postoperative antibiotics should be reserved for specific indications, for example when a surgical site becomes contaminated in an enucleation, when removing an eye with an infectious keratitis or in case of relevant comorbidities of the patient.

Following keratectomy 80% of veterinarians use antibiotics. When performing a keratectomy the diseased cornea may or may not be vascularized, which is likely to influence whether or not a systemic antibiotic reaches the ocular surface.

Amoxicillin with clavulanic acid was the only systemic antibiotic used in keratecomies. No studies are available on the pharmokinetics of amoxicillin related to the ocular and periocular tissues or its penetration into the tear film. For other antibiotics including doxycycline and ceftiofur it has been shown that the tear film is reached following oral administration, but without reliably achieving minimal inhibitory concentrations (MIC) for relevant bacteria.^34^, ^35^, ^36^ This discrepancy shows how important it is to investigate pharmacokinetics before using antibiotics for unusual anatomic locations such as the avascular cornea.

Given the potentially devastating consequences of an infectious keratitis, topical postoperative antibiotics are considered essential in human keratorefractive surgery.^37^ When using antibiotics topically the challenges are the same as in conjunctival surgery. If topical antibiotics do not measurably change the composition of the flora, how do they reliably prevent SSIs? Compared to the aforementioned soft tissue surgeries, it is a unique feature of the keratectomy that it may take several days for the cornea to epithelialize, leaving the stroma exposed. This poses a risk, especially since corneal infections can have a long‐term detrimental effect on corneal clarity or may even result in the loss of an eye if an infectious keratitis occurs.^38^, ^39^ Monitoring wound healing to detect complications early is essential. The use of prophylactic treatment to prevent SSIs currently appears to be indicated. While antibiotics are the obvious choice postulated for many years, it is worth looking at alternatives.

A number of antiseptic agents are available for use on the ocular surface. Polyhexanid, hypochoric acid, povidone iodine, and N‐acetylcysteine show antimicrobial effects against the three species most commonly involved in infectious keratitis in dogs and cats, namely *Staphylococcus* spp., *Streptococcus* spp. *and Pseudomonas* spp.^40^, ^41^ These antiseptics are more likely to address the overall bacterial load, rather than selecting against sensitive species. N‐acetylcysteine with a comparably mild but broad antimicrobial effect has the additional benefits of inhibiting collagenases and biofilm formation making it an interesting alternative, without the disadvantage of creating antimicrobial resistances.^40^, ^42^, ^43^ Multi‐centered prospective studies are warranted to further test if these antiseptics are more likely to prevent corneal SSIs especially when faced with multidrug‐resistant bacteria. Cleaning the ocular surface once with povidone iodine (1:50) reduced the bacterial load, but did not change the overall composition of the microbiome long‐term.^28^ Detrimental effects of antiseptics on the bacterial flora following their use over several days will need to be investigated.

This survey highlights a clear discrepancy between evidence of prudent antimicrobial use and the daily practice in ophthalmic soft tissue surgery in Germany. This discrepancy may in part be explained by the fact that fear of complications and personal experience are common drivers when choosing to use antibiotics. Reported barriers to rational antibiotic use in general include internal barriers like knowledge and attitude as well as external barriers like social pressure and unclear instructions.^44^ Interestingly, despite global concerns surrounding antimicrobial resistance^3^; the majority of veterinary surgeons in Germany are not concerned about the development of antimicrobial resistance. Furthermore, the expression “never change a winning team” is common reasoning to keep current practices even though this may mean preventing change toward an equally good or better alternative treatment choice.^44^ To overcome these barriers, evidence alone is not enough. It is a valid concern that patients may be hurt if adequate precautions are not taken, especially if traditional treatment protocols appear to be successful by one's own experience. Evidence based guidelines for a good antimicrobial stewardship help veterinarians to change behavior toward a more restrictive use of antimicrobials.^45^ Our study also showed that the presence of guidelines on antimicrobial stewardship meant that veterinary surgeons were less likely to use postoperative antibiotics in enucleations or to choose topical fluoroquinolones following eyelid surgery.

## 5 CONCLUSION

Most veterinarians in Germany opt for postoperative antibiotics following ophthalmic procedures, including enucleation, eyelid, and TEL surgery, as well as keratectomy. This choice seems to be influenced by individual clinical experience and concerns about potential complications. However, this practice stands in contrast to the emerging body of evidence suggesting that postoperative antibiotics may not offer significant benefits in preventing SSIs in clean soft tissue surgery. This should also apply to ophthalmic procedures, possibly with the exception of corneal wounds that take several days to close. The concern about antimicrobial resistance amongst the participating veterinarians is low, despite rising global concerns. To address prevailing barriers impeding the prudent use of antibiotics, we therefore propose a collaborative effort led by the Colleges for Veterinary Ophthalmology with the aim to coordinate the research, to fill evidence gaps and to create a consensus statement promoting the responsible use of antibiotics in veterinary ophthalmology, in line with the One Health approach.

## AUTHOR CONTRIBUTIONS

**Claudia Busse:** Conceptualization; formal analysis; methodology; resources; supervision; writing – original draft. **Anne Raab:** Formal analysis; investigation. **Lothar Kreienbrock:** Formal analysis; writing – review and editing. **Holger Andreas Volk:** Funding acquisition; supervision; writing – review and editing.

## CONFLICT OF INTEREST STATEMENT

The authors declare no conflict of interest.

## ETHICAL STATEMENT

This study is exempt from an institutional review board approval. The data recruitment and the questionnaire complied with the current Data Protection Directive of the European Union and Germany and was authorized by the data protection officer of the University of Veterinary Medicine Hannover Foundation.

## Data Availability

The data that support the findings of this study are available from the corresponding author upon reasonable request.
